# Supplementary material for: Isolation, diversity, and antimicrobial activity of fungal endophytes from Rohdea chinensis (Baker) N.Tanaka (synonym Tupistra chinensis Baker) of Qinling Mountains, China
Source: PeerJ. 2020 Jun 17;8:e9342. doi: 10.7717/peerj.9342 (PMC7305772; doi:10.7717/peerj.9342)
Supplement: File S2 [file peerj-08-9342-s002.pdf]

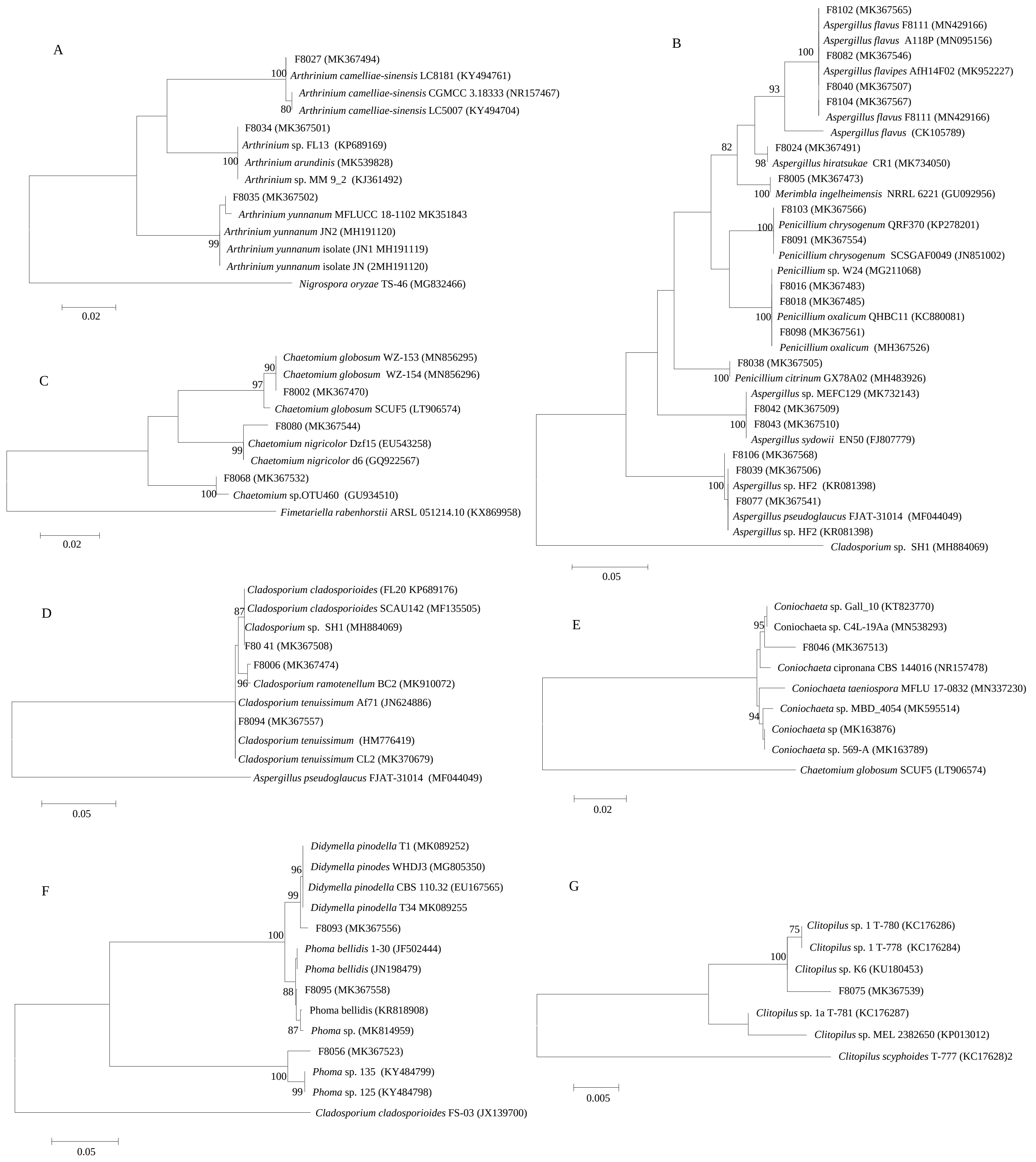
A
F8027 (MK367494)
100
Arthrinium camelliae-sinensis LC8181 (KY494761)
Arthrinium camelliae-sinensis CGMCC 3.18333 (NR157467)
80
Arthrinium camelliae-sinensis LC5007 (KY494704)
F8034 (MK367501)
Arthrinium sp. FL13 (KP689169)
100
Arthrinium arundinis (MK539828)
Arthrinium sp. MM 9_2 (KJ361492)
F8035 (MK367502)
Arthrinium yunnanum MFLUCC 18-1102 MK351843
Arthrinium yunnanum JN2 (MH191120)
99
Arthrinium yunnanum isolate (JN1 MH191119)
Arthrinium yunnanum isolate JN (2MH191120)
Nigrospora oryzae TS-46 (MG832466)
0.02
B
F8102 (MK367565)
Aspergillus flavus F8111 (MN429166)
Aspergillus flavus A118P (MN095156)
100
F8082 (MK367546)
Aspergillus flavipes AfH14F02 (MK952227)
93
F8040 (MK367507)
F8104 (MK367567)
Aspergillus flavus F8111 (MN429166)
Aspergillus flavus (CK105789)
82
F8024 (MK367491)
98
Aspergillus hiratsukae CR1 (MK734050)
F8005 (MK367473)
100
Merimbla ingelheimensis NRRL 6221 (GU092956)
F8103 (MK367566)
100
Penicillium chrysogenum QRF370 (KP278201)
F8091 (MK367554)
Penicillium chrysogenum SCSGAF0049 (JN851002)
Penicillium sp. W24 (MG211068)
F8016 (MK367483)
F8018 (MK367485)
100
Penicillium oxalicum QHBC11 (KC880081)
F8098 (MK367561)
Penicillium oxalicum (MH367526)
F8038 (MK367505)
100
Penicillium citrinum GX78A02 (MH483926)
Aspergillus sp. MEFC129 (MK732143)
F8042 (MK367509)
100
F8043 (MK367510)
Aspergillus sydowii EN50 (FJ807779)
F8106 (MK367568)
F8039 (MK367506)
100
Aspergillus sp. HF2 (KR081398)
F8077 (MK367541)
Aspergillus pseudoglaucus FJAT-31014 (MF044049)
Aspergillus sp. HF2 (KR081398)
Cladosporium sp. SH1 (MH884069)
0.05
C
90
Chaetomium globosum WZ-153 (MN856295)
Chaetomium globosum WZ-154 (MN856296)
97
F8002 (MK367470)
Chaetomium globosum SCUF5 (LT906574)
F8080 (MK367544)
Chaetomium nigricolor Dzf15 (EU543258)
99
Chaetomium nigricolor d6 (GQ922567)
F8068 (MK367532)
100
Chaetomium sp.OTU460 (GU934510)
Fimetariella rabenhorstii ARSL 051214.10 (KX869958)
0.02
D
Cladosporium cladosporioides (FL20 KP689176)
87
Cladosporium cladosporioides SCAU142 (MF135505)
Cladosporium sp. SH1 (MH884069)
F80 41 (MK367508)
F8006 (MK367474)
96
Cladosporium ramotenellum BC2 (MK910072)
Cladosporium tenuissimum Af71 (JN624886)
F8094 (MK367557)
Cladosporium tenuissimum (HM776419)
Cladosporium tenuissimum CL2 (MK370679)
Aspergillus pseudoglaucus FJAT-31014 (MF044049)
0.05
E
Coniochaeta sp. Gall_10 (KT823770)
95
Coniochaeta sp. C4L-19Aa (MN538293)
F8046 (MK367513)
Coniochaeta cipronana CBS 144016 (NR157478)
Coniochaeta taeniospora MFLU 17-0832 (MN337230)
Coniochaeta sp. MBD_4054 (MK595514)
94
Coniochaeta sp (MK163876)
Coniochaeta sp. 569-A (MK163789)
Chaetomium globosum SCUF5 (LT906574)
0.02
F
Didymella pinodella T1 (MK089252)
96
Didymella pinodes WHDJ3 (MG805350)
Didymella pinodella CBS 110.32 (EU167565)
99
Didymella pinodella T34 MK089255
F8093 (MK367556)
100
Phoma bellidis 1-30 (JF502444)
Phoma bellidis (JN198479)
88
F8095 (MK367558)
Phoma bellidis (KR818908)
87
Phoma sp. (MK814959)
F8056 (MK367523)
100
Phoma sp. 135 (KY484799)
99
Phoma sp. 125 (KY484798)
Cladosporium cladosporioides FS-03 (JX139700)
0.05
G
75
Clitopilus sp. 1 T-780 (KC176286)
Clitopilus sp. 1 T-778 (KC176284)
100
Clitopilus sp. K6 (KU180453)
F8075 (MK367539)
Clitopilus sp. 1a T-781 (KC176287)
Clitopilus sp. MEL 2382650 (KP013012)
Clitopilus scyphoides T-777 (KC17628)2
0.005

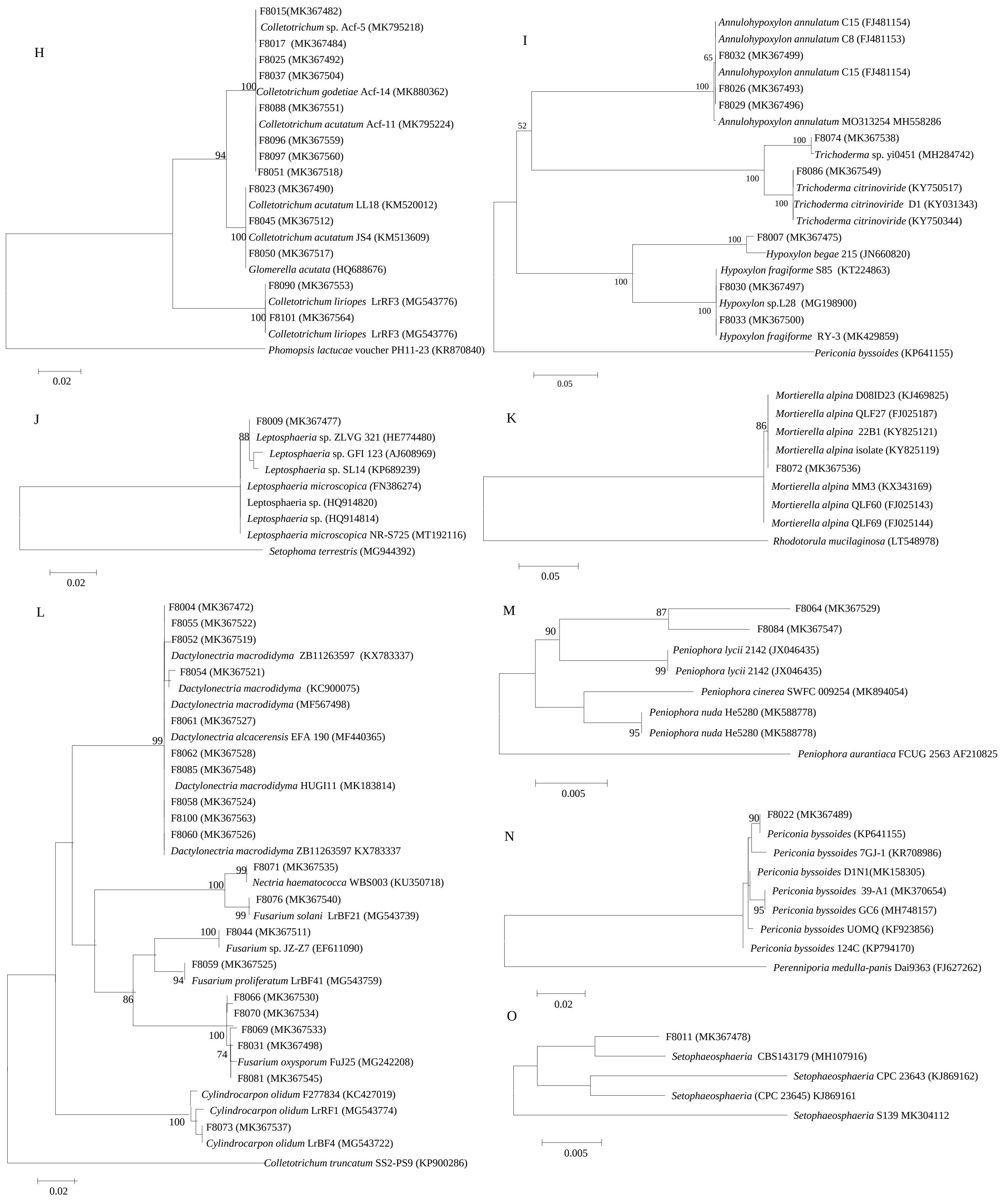

H
F8015(MK367482)
Colletotrichum sp. Acf-5 (MK795218)
F8017 (MK367484)
F8025 (MK367492)
F8037 (MK367504)
100
Colletotrichum godetiae Acf-14 (MK880362)
F8088 (MK367551)
Colletotrichum acutatum Acf-11 (MK795224)
F8096 (MK367559)
F8097 (MK367560)
94
F8051 (MK367518)
F8023 (MK367490)
Colletotrichum acutatum LL18 (KM520012)
F8045 (MK367512)
100
Colletotrichum acutatum JS4 (KM513609)
F8050 (MK367517)
Glomerella acutata (HQ688676)
F8090 (MK367553)
Colletotrichum liriopes LrRF3 (MG543776)
100
F8101 (MK367564)
Colletotrichum liriopes LrRF3 (MG543776)
Phomopsis lactucae voucher PH11-23 (KR870840)
0.02
I
Annulohypoxylon annulatum C15 (FJ481154)
Annulohypoxylon annulatum C8 (FJ481153)
65
F8032 (MK367499)
Annulohypoxylon annulatum C15 (FJ481154)
100
F8026 (MK367493)
F8029 (MK367496)
52
Annulohypoxylon annulatum MO313254 MH558286
100
F8074 (MK367538)
Trichoderma sp. yi0451 (MH284742)
F8086 (MK367549)
100
Trichoderma citrinoviride (KY750517)
100
Trichoderma citrinoviride D1 (KY031343)
Trichoderma citrinoviride (KY750344)
100
F8007 (MK367475)
Hypoxylon begae 215 (JN660820)
Hypoxylon fragiforme S85 (KT224863)
100
F8030 (MK367497)
Hypoxylon sp.L28 (MG198900)
100
F8033 (MK367500)
Hypoxylon fragiforme RY-3 (MK429859)
Periconia byssoides (KP641155)
0.05
J
F8009 (MK367477)
88
Leptosphaeria sp. ZLVG 321 (HE774480)
Leptosphaeria sp. GFI 123 (AJ608969)
Leptosphaeria sp. SL14 (KP689239)
Leptosphaeria microscopica (FN386274)
Leptosphaeria sp. (HQ914820)
Leptosphaeria sp. (HQ914814)
Leptosphaeria microscopica NR-S725 (MT192116)
Setophoma terrestris (MG944392)
0.02
K
Mortierella alpina D08ID23 (KJ469825)
Mortierella alpina QLF27 (FJ025187)
86
Mortierella alpina 22B1 (KY825121)
Mortierella alpina isolate (KY825119)
F8072 (MK367536)
Mortierella alpina MM3 (KX343169)
Mortierella alpina QLF60 (FJ025143)
Mortierella alpina QLF69 (FJ025144)
Rhodotorula mucilaginosa (LT548978)
0.05
L
F8004 (MK367472)
F8055 (MK367522)
F8052 (MK367519)
Dactylonectria macrodidyma ZB11263597 (KX783337)
F8054 (MK367521)
Dactylonectria macrodidyma (KC900075)
Dactylonectria macrodidyma (MF567498)
F8061 (MK367527)
99
Dactylonectria alcacerensis EFA 190 (MF440365)
F8062 (MK367528)
F8085 (MK367548)
Dactylonectria macrodidyma HUGI11 (MK183814)
F8058 (MK367524)
F8100 (MK367563)
F8060 (MK367526)
Dactylonectria macrodidyma ZB11263597 KX783337
99
F8071 (MK367535)
100
Nectria haematococca WBS003 (KU350718)
F8076 (MK367540)
99
Fusarium solani LrBF21 (MG543739)
100
F8044 (MK367511)
Fusarium sp. JZ-Z7 (EF611090)
F8059 (MK367525)
94
Fusarium proliferatum LrBF41 (MG543759)
86
F8066 (MK367530)
F8070 (MK367534)
F8069 (MK367533)
100
F8031 (MK367498)
74
Fusarium oxysporum FuJ25 (MG242208)
F8081 (MK367545)
Cylindrocarpon olidum F277834 (KC427019)
Cylindrocarpon olidum LrRF1 (MG543774)
100
F8073 (MK367537)
Cylindrocarpon olidum LrBF4 (MG543722)
Colletotrichum truncatum SS2-PS9 (KP900286)
0.02
M
87
F8064 (MK367529)
90
F8084 (MK367547)
Peniophora lycii 2142 (JX046435)
99
Peniophora lycii 2142 (JX046435)
Peniophora cinerea SWFC 009254 (MK894054)
Peniophora nuda He5280 (MK588778)
95
Peniophora nuda He5280 (MK588778)
Peniophora aurantiaca FCUG 2563 AF210825
0.005
N
90
F8022 (MK367489)
Periconia byssoides (KP641155)
Periconia byssoides 7GJ-1 (KR708986)
Periconia byssoides D1N1(MK158305)
Periconia byssoides 39-A1 (MK370654)
95
Periconia byssoides GC6 (MH748157)
Periconia byssoides UOMQ (KF923856)
Periconia byssoides 124C (KP794170)
Perenniporia medulla-panis Dai9363 (FJ627262)
0.02
O
F8011 (MK367478)
Setophaeosphaeria CBS143179 (MH107916)
Setophaeosphaeria CPC 23643 (KJ869162)
Setophaeosphaeria (CPC 23645) KJ869161
Setophaeosphaeria S139 MK304112
0.005

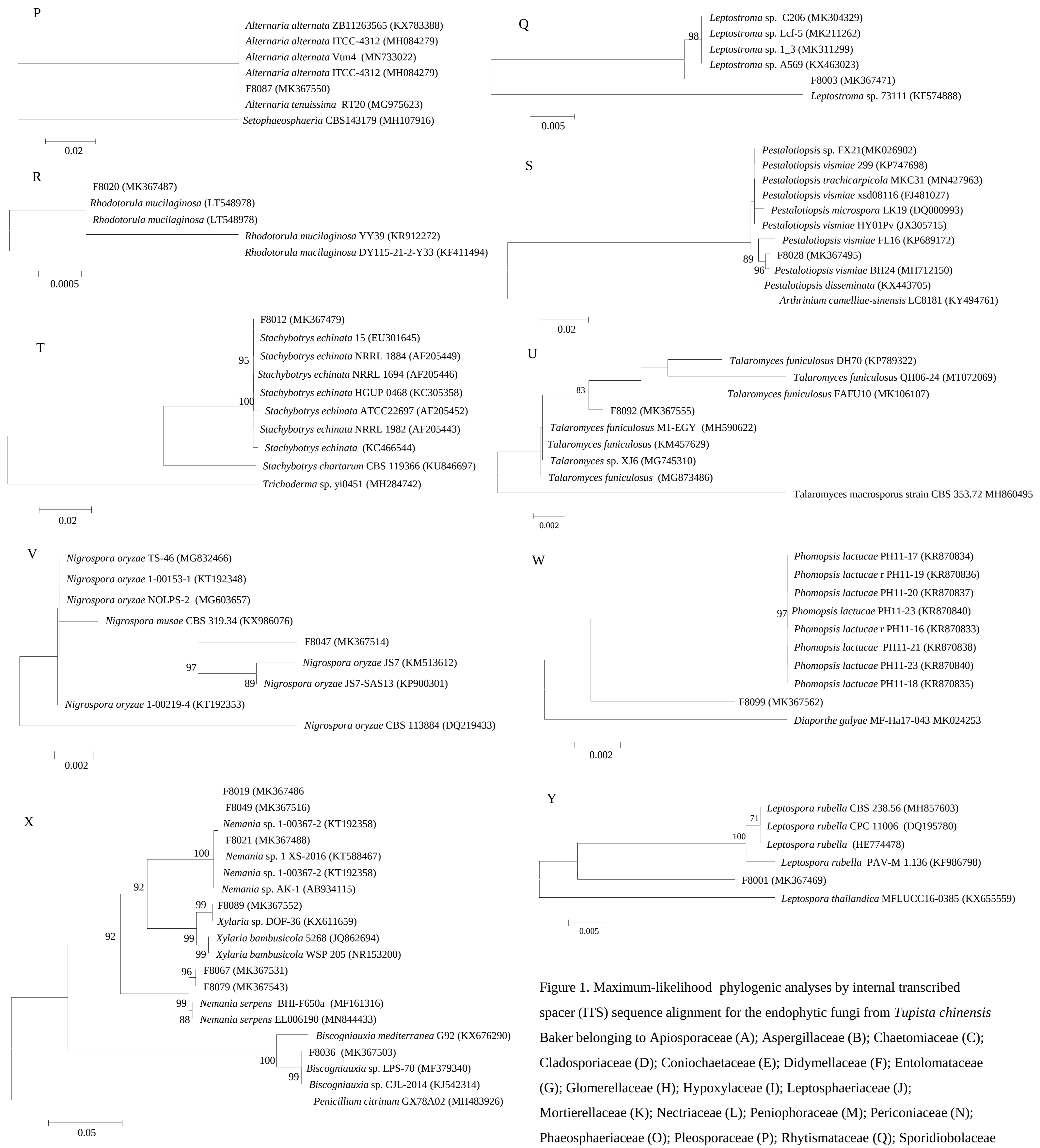

Figure 1. Maximum-likelihood phylogenic analyses by internal transcribed spacer (ITS) sequence alignment for the endophytic fungi from *Tupista chinensis* Baker belonging to Apiosporaceae (A); Aspergillaceae (B); Chaetomiaceae (C); Cladosporiaceae (D); Coniochaetaceae (E); Didymellaceae (F); Entolomataceae (G); Glomerellaceae (H); Hypoxylaceae (I); Leptosphaeriaceae (J); Mortierellaceae (K); Nectriaceae (L); Peniophoraceae (M); Periconiaceae (N); Phaeosphaeriaceae (O); Pleosporaceae (P); Rhytismataceae (Q); Sporidiobolaceae (R); Sporocadaceae (S); Stachybotryaceae (T); Trichocomaceae (U); Trichosphaeriaceae (V); Valsaceae (W); Xylariaceae (X) and incertae sedis (Y).
